# Effects and action mechanisms of lotus leaf (*Nelumbo nucifera*) ethanol extract on gut microbes and obesity in high-fat diet-fed rats

**DOI:** 10.3389/fnut.2023.1169843

**Published:** 2023-06-26

**Authors:** Zhang Yanan, Ma Lu, Zhang Lu, Huo Jinhai, Wang Weiming

**Affiliations:** ^1^Heilongjiang Academy of Chinese Medicine Science, Institute of Chinese Materia Medica, Harbin, China; ^2^Institute of Chinese Materia Medica, Heilongjiang Nursing College, Harbin, China

**Keywords:** lotus leaf ethanol extract, intestinal flora, inflammatory factor, obesity, lipid metabolism

## Abstract

**Objective:**

The present study aimed to clarify the effect of the lotus leaf ethanol extract (LLEE) on the mechanism of antiobesity and the intestinal microbiota of obese rats.

**Methods:**

A total of 40 specific pathogen-free (SPF) male Sprague–Dawley (SD) rats were split into the blank control group, the model control group, the Orlistat capsule control group, and the LLEE group. All the groups were intervened and fed specific diets for 5 months. During the experiment, we evaluated the rats' body weight, length, serum biochemical indicators, and inflammatory factor levels. After dissection, the liver; epididymal and perirenal white adipose tissue (WAT); and the contents of the cecum were collected for pathological evaluation and intestinal flora analysis.

**Results:**

Lotus leaf alcohol extract can significantly reduce the serum total cholesterol, triglyceride, and low-density lipoprotein cholesterol levels. It also decreases the accumulation of fatty deposits in the liver of rats and the levels of serum inflammatory factors IL-6 and TNF-α and increases the level of IL-10. Lotus leaf alcohol extracts significantly increased the abundance of *Muribaculaceae* in the intestinal flora of rats, reduced the abundance of pro-inflammatory bacteria *Firmicutes*, and relieved fatty liver and other inflammation and diseases caused by a high-fat diet. Besides, the ethanol extract of the lotus leaf significantly regulated the abundance of *Ruminococcus*, suggesting that the ethanol extract of the lotus leaf may prevent hyperlipidemia.

**Conclusion:**

We elucidated the effects and action mechanisms of LLEE on obesity in high-fat diet-fed rats to provide suggestions for regulating intestinal flora through dietary intervention and thus improving blood lipid metabolism.

## Introduction

Obesity (1, 2) is caused by the excessive accumulation of body fat due to poor diet, lack of exercise, and other reasons, which are the basis of many diseases. With the improvement of people's living standards and the change in lifestyle, the incidence of obesity is getting higher and higher. Orlistat capsule (3, 4), as a well-known drug, is suitable for the long-term treatment of obesity and overweight patients with moderate dietary control and exercise, including patients who have already presented with risk factors related to obesity. It serves as a medication control for the effect of lotus leaf ethanol extract (LLEE) in this study.

Lotus leaf, as a homologous substance of food and medicine, has potential advantages in regulating obesity (5). Its regulatory mechanism is characterized by multiple targets, stable efficacy, and high safety. Modern pharmacological studies have shown that lotus leaf extract can reduce the digestive capacity of the body, reduce the absorption of lipids and carbohydrates, and regulate energy consumption so as to effectively improve hyperlipidemia and obesity (6–8). The study of intestinal flora has become a hot topic in the field of medicine, and its composition is affected by genetics, diet, body weight, drugs, and host metabolic state (9, 10). Currently, few studies have shown whether there is a correlation between intestinal microbes and diseases (11, 12). The purpose of this study was to clarify the interaction between obesity caused by a high-fat diet and intestinal flora so as to provide an experimental basis for the development of new weight-loss products.

## 2. Materials and methods

### 2.1. Animals, materials, and reagents

Sprague–Dawley rats [Animal certificate: SCXK (H) 2019-001, HMU Animal Experiments Center]; Maintain Feed (production license No. 1016706714625204224, Beijing Keao–Xieli Feed Co., Ltd); High-fat feed (production license No. 1016712400381763584, Beijing Keao–Xieli Feed Co., Ltd); Orlistat capsules (Zhienyiyao Co., Ltd, Chongqing); Lotus Leaf (Beijing Tong Ren Tang Co., Ltd, Haerbin). Referring to the method discussed by Yan and Jun (13), raw lotus leaf products were crushed and sieved through a 20-mesh sieve to obtain lotus leaf powder. Then, we added 70% ethanol solution to the powder in a ratio of 40:1, and the mixture was soaked at room temperature for 30 min, ultrasonicated for 40 min (frequency 500 kW, 35°C), recovered by rotary evaporator ethanol, evaporated to dryness to obtain the extract, and stored in a refrigerator at 4°C for use.

A total cholesterol (TC) assay kit, triglyceride (TG) test kit, low-density lipoprotein cholesterol (LDL-C) test kit, rat interleukin-6 (IL-6) ELISA kit, rat interleukin-10 (IL-10) ELISA kit, and rat tumor necrosis factor-α (TNF-α) enzyme-linked immunoassay kit were obtained from Nanjing Jiancheng Bioengineering Institute, China and fecal total DNA extraction kit from Tiangen Bio, China.

### 2.2. Instruments and equipment

The following instruments were used: Phusion High-Fidelity PCR (Biolabs, New England), Master Mix with GC Buffer (Biolabs, New England), MiSeq Reagent Kit (Illumina, USA), Phusion High-Fidelity DNA polymerase (Biolabs, New England), Library Preparation Kit (Illumina, USA), Ultralow temperature refrigerator (SANYO Company, Japan), refrigerated high-speed centrifuge (Thermo Fisher Scientific, Inc., USA), Infinite M200 PRO microplate reader (Tecan, Switzerland), T100 Thermal Cycler (Bio-rad, USA), and Illumina NovaSeq 6000 Sequencing Platform (Illumina, USA).

### 2.3. Methods

#### 2.3.1. Animal grouping and feeding

In this experiment, 40 SPF male SD rats were bred under an environment of a temperature of 22 ± 2°C; relative humidity of 50 ± 20%; and 12-h light–dark cycles. After a week of adaptive feeding, they were randomly and equally divided into four groups according to body weight (BW) and were given 5-month intervening feeding. The blank control group (BC) was fed with conventional feed, and the model control group (MC), Orlistat capsules control group (OC), and the lotus leaf extract group (LLEE) were fed with high-fat feed. The BC group and the MC group were given water daily. Rats in the OC group were fed an Orlistat capsule water solution at a dose of 0.035 mg/kg, and rats in the LLEE group were fed an LLEE water solution at a dose of 1,050 g/kg daily. According to the BW, each group was given a ratio of l ml/l00 g intragastric administration once. The same animals (i.e., OC and LLEE groups), in addition to intragastric administration o.d. Orlistat or LLEE, were allowed to eat and drink freely. All animals were weighed and recorded each month.

#### 2.3.2. Sample collection and processing

During the experiment, intraocular canthal blood (1.5 ml) was taken from rats at the first, second, third, fourth, and fifth months of the intervention. The supernatant was centrifuged and stored as serum samples in the refrigerator at −20°C. After the experiment, the rat cecal contents were collected aseptically and stored in a −80°C freezer. After the last administration, each group of rats fasted for 12 h, and ~2 ml of blood was collected from the abdominal aorta at 3,000 rpm/min at 4°C and centrifuged for 10 min to obtain serum in an ultralow temperature freezer at −80°C for use. After collecting blood, the liver was stripped and weighed, and the liver with a volume of 1 cm^3^ was placed in 4% paraformaldehyde fixative solution and stored in a refrigerator at 4°C. WAT of epididymal and perirenal was separated and weighed.

### 2.4. Determination of relevant indexes in rats

#### 2.4.1. General signs

The body weight and length of rats were recorded each month to calculate the Lee index [= ^3^√body weight (g)/naso–anal length (cm)]. Epididymal and perirenal WATs were weighed to calculate the percentage of visceral fat coefficient [= (perirenal fat mass + epididymal fat mass)/body weight].

#### 2.4.2. Serum indexes of rats

Serum samples of rats were used to measure serum biochemical markers and inflammatory factors levels. Frozen serum samples were defrosted at room temperature for 30 min, and 100 μl of serum was drawn from a pipette. According to the instructions of the kit, the level of serum total cholesterol was measured by the CHOD-PAP method (Roche P-Modular), triglycerides were measured by the GPO-PAP method (Roche P-Modular), and low-density lipoprotein cholesterol was obtained by the Friedewald formula. The contents of IL-6, IL-10, and TNF-α were detected by enzyme-linked immunosorbent assay (ELISA).

#### 2.4.3. Morphology of rat liver

The liver tissues of rats were cut into 1-cm^3^ pieces, fixed with 10% neutral formaldehyde, and dehydrated with gradient ethanol: 75% ethanol, 90 min; 95% ethanol, 90 min; 100% ethanol, 90 min, 3 times; and 100% xylene, 60 min, 2 times. Then, they were soaked with paraffin wax two times for 2 h. After embedding, sections were sliced into 6-μm pieces and placed on slides for hematoxylin and eosin (HE) staining and optical microscope observation.

#### 2.4.4. Sequencing of rat cecal contents

A certain amount of frozen stored fecal samples were thawed at room temperature for 30 min; DNA was extracted according to the instructions of the soil genomic DNA intense extraction kit and stored at −20°C. Amplification primers (338F5′-ACTCCTACGGGAGGCAGCA-3′ and 806R5′GGACTACHVGGGTWTCTAAT-3′) were used to amplify the V3–V4 regions of 16SrDNA. High-throughput sequencing of the V3–V4 region of 16SrDNA was performed using the Illumina Hiseq 2500 platform and QIIMEv1.8.0 software (Beijing Bimai Ke Biotechnology Co., Ltd, Beijing). The VSEARCH 2.8.1 software was used to merge the high-throughput sequencing data, remove primers, and eliminate redundancy. The usearch10 software was used to denoise the sequence exact sequence variant (ESVs). Referring to the Silva database, operational taxonomic units (OTUs) were obtained by clustering at a 97% similarity level. The NovoMagic analysis platform was used to construct and analyze the results of intestinal flora, and OTU clustering, species annotation, sample complexity analysis, and multisampling comparative analysis were performed.

### 2.5. Statistical analysis

All data were analyzed and processed using the SPSS Statistics 25.0 statistical software (IBM). The test data were expressed as mean ± standard deviation. One-way analysis of variance (ANOVA) was used for the mean comparison among multiple groups, and the LSD method was used for further pair comparison. A *p*-value of < 0.05 was considered a statistically significant difference.

## 3. Results and analysis

### 3.1. General observation

During the experiment, all groups of rats were allowed to eat and drink freely. Except for bloating, diarrhea, and the dull fur color appearance of animals in the model group, animals in the other groups were generally in good condition with no abnormal signs.

### 3.2. Effects of LLEE on BW of rats

**Figure 1:**
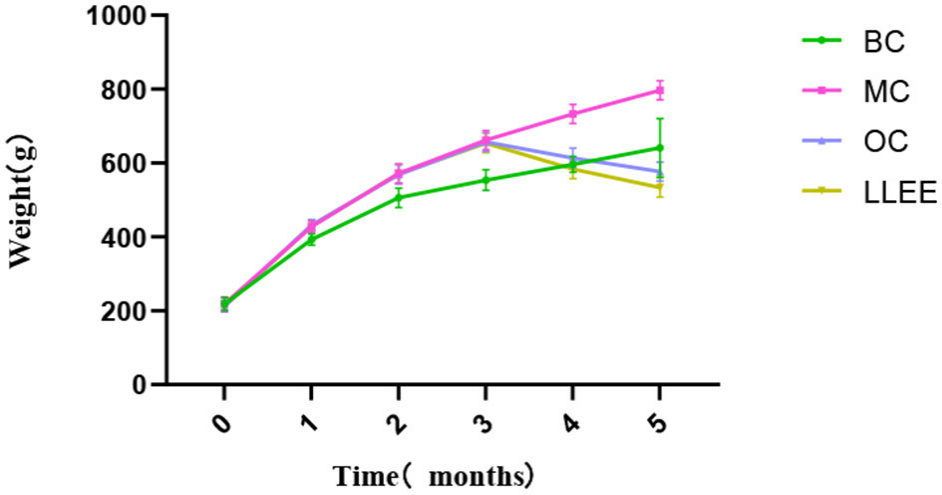
Body weight trends of rats in each group.

Presently, there is no unified standard for high-fat diet-induced obesity models in rats, and a large number of relevant studies take the body weight of the MC group as the evaluation standard, which is 20% higher than that of the BC group (14). There was no significant difference in initial body mass among all groups (*p* > 0.05). The body weight recorded during the experiment (Table 1) showed that the weight of the BC, OC, and LLEE groups was significantly lower than that of the MC group (*p* < 0.01), while there was no statistical difference between the OC and LLEE groups (*p* > 0.05) (Figure 1).

**Table 1:** Body weight levels of 0–5 months of rats in each group (g).

**Months**	**BC (*N* = 10)**	**MC (*N* = 10)**	**OC (*N* = 10)**	**LLEE (*N* = 10)**
0	218.50 ± 17.41	218.50 ± 18.73	213.30 ± 16.57	212.30 ± 14.43
1	392.70 ± 14.97	427.70 ± 14.97	431.70 ± 14.97	429.70 ± 14.97
2	506.00 ± 26.13	572.00 ± 26.13	569.00 ± 26.13	571.00 ± 26.13
3	554.10 ± 28.14	573.90 ± 30.93	573.56 ± 25.71	582.75 ± 16.53
4	596.60 ± 21.33	610.50 ± 13.49	563.51 ± 23.54	549.93 ± 16.20
5	641.20 ± 79.36	678.40 ± 22.97	547.54 ± 18.66	511.44 ± 11.74

### 3.3. Effects of LLEE on the Lee index in rats

**Figure 2:**
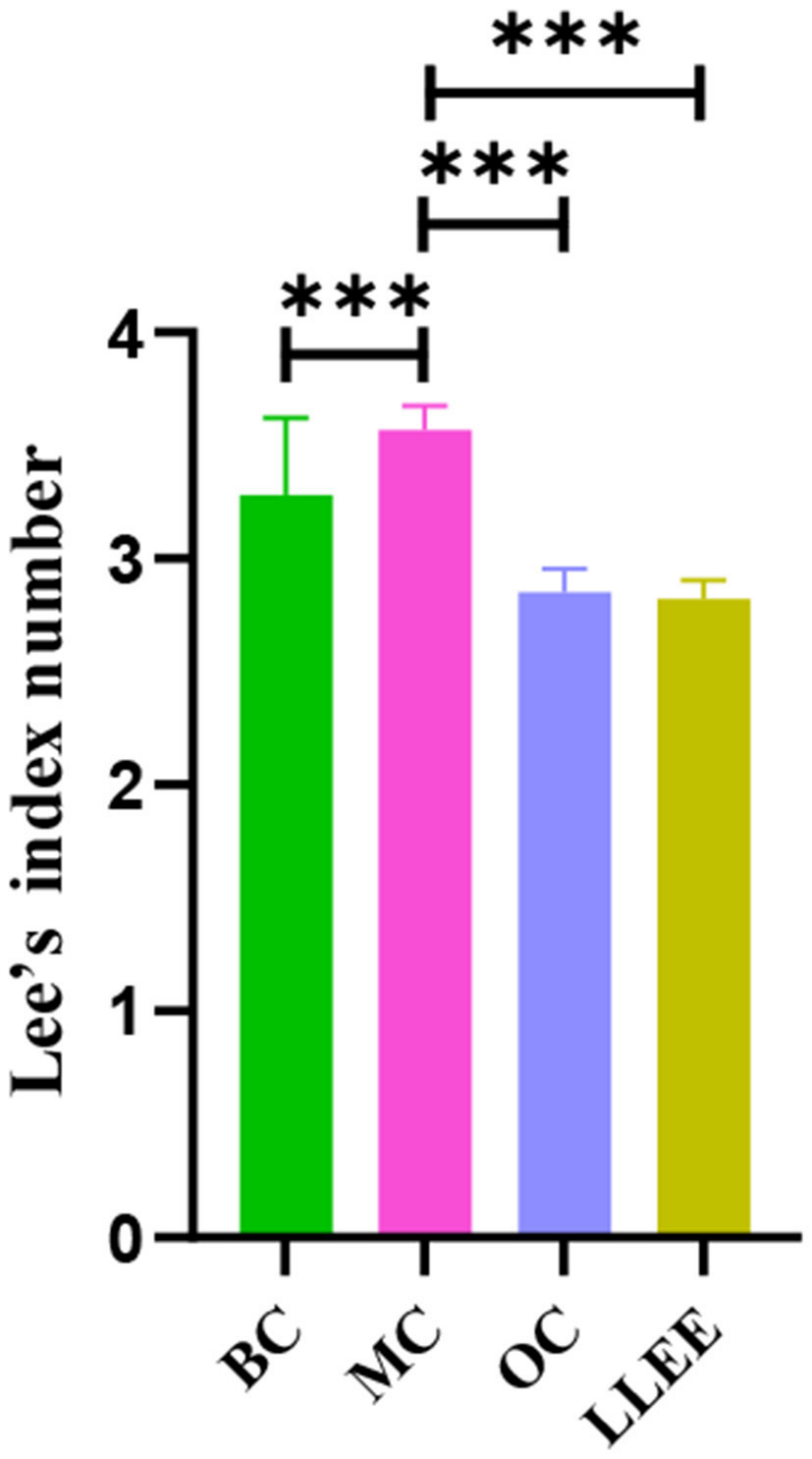
The Lee index of rats in each group. ^***^P < 0.001.

Presently, the Lee index is an effective index to evaluate the degree of obesity. In general, the higher the Lee index, the greater the degree of obesity. The Lee index of the OC, LLEE, and BC groups was significantly different from that of the MC group (*p* < 0.01), while there was no significant difference between the Lee index of the OC and LLEE groups (*p* > 0.05) (Figure 2).

### 3.4. Effect of LLEE on visceral fat coefficient in rats

**Figure 3:**
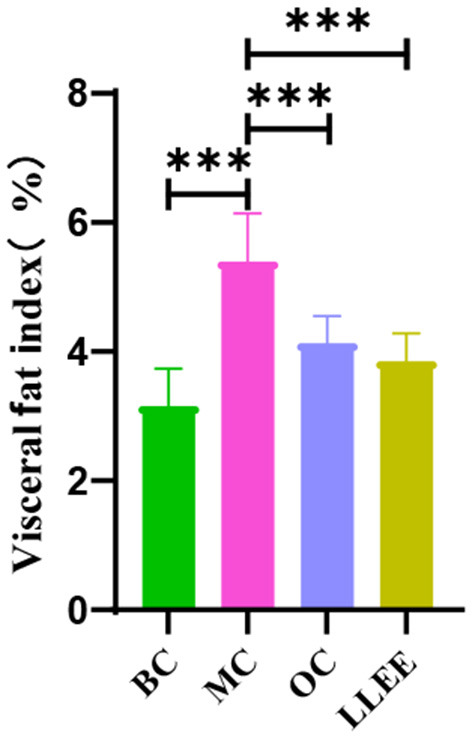
The percentage of visceral fat coefficient of rats in each group. ^***^P < 0.001.

WAT is widely distributed around the subcutaneous tissue and viscera in the body. The main function of WAT is to store excess energy in the body in the form of neutral fat. In Figure 3, there was a significant difference (*p* < 0.01) in the visceral WAT ratio between the MC group and the BC group (*p* < 0.01), indicating that the high-fat diet successfully induced obesity in rats. Compared with the BC group, the LLEE group and the OC group showed significant intergroup differences (*p* < 0.01 and *p* < 0.05, respectively), which proved that LLEE had a more significant effect on reducing visceral WAT than the Orlistat capsule.

### 3.5. Effects of LLEE on liver tissue morphology

**Figure 4:**
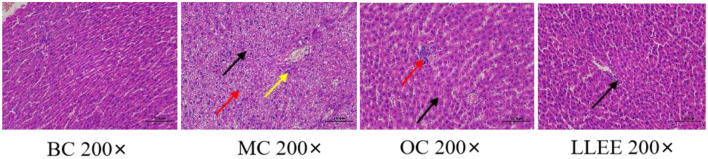
The liver tissue morphology of rats.

As shown in Figure 4, the liver tissue of rats in the BC group showed that the central lobule of the liver was the central vein, surrounded by hepatocytes and hepatic sinuses arranged in a roughly radial arrangement, and the hepatocytes were round and full. The liver plates were arranged regularly and neatly, and the hepatic sinuses were not significantly dilated or squeezed. There were no obvious abnormalities in the portal area between adjacent hepatic lobules. No significant abnormalities were observed. The liver tissue of rats in the MC group was widely observed with hepatocyte steatosis, tiny circular vacuoles (black arrows) in the cytoplasm, a large number of hepatocytes accompanied by balloon-like changes, swollen cells, vacuole-like cytoplasm (red arrows), and multiple small focal infiltrates of inflammatory cells (yellow arrows) in lobules and around veins. A small number of liver cells with mild steatosis were observed in the liver tissue of rats in the OC group, tiny circular vacuoles (black arrows) could be noted in the cytoplasm, and several small focal infiltrates of inflammatory cells (red arrows) could also be observed around the veins and in the lobules. Mild congestion of the hepatic sinuses in the LLEE group (black arrows) was noticed. Thus, compared with the Orlistat capsule, LLEE can significantly improve the morphology of liver cells and reduce the infiltration of inflammatory cells such as liver lobules.

### 3.6. Effects of LLEE on lipid levels in rats

**Figure 5:**
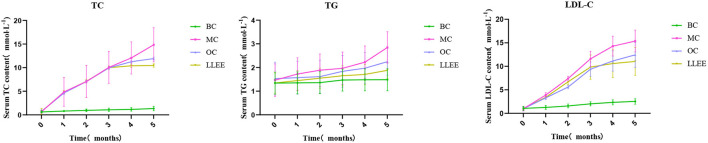
The trends of TC, TG, and LDL-C of rats in each group.

The contents of TC, TG, and LDL-C in the serum of rats in each group were changed in the intervention stage, as shown in Figure 5. At the beginning of the experiment, there were no significant differences in the contents of TC, TG, and LDL-C in the serum of rats among all groups (*p* > 0.01). During the intervention, the serum biochemical indices of the BC group remained stable, while the serum TC and LDL-C contents of the MC, OC, and LLEE groups increased rapidly with the increase of feeding time. At the end of the intervention, the serum biochemical indices of the three groups were significantly different from those of the BC group, indicating that continuous feeding of a high-fat diet could lead to abnormal serum biochemical indices of the rats. Compared with the OC group, the serum biochemical indices in the LLEE group showed a similar increasing or decreasing trend, and the effect was more obvious (*p* < 0.05).

### 3.7. Effects of LLEE on serum inflammatory factors in rats

**Figure 6:**
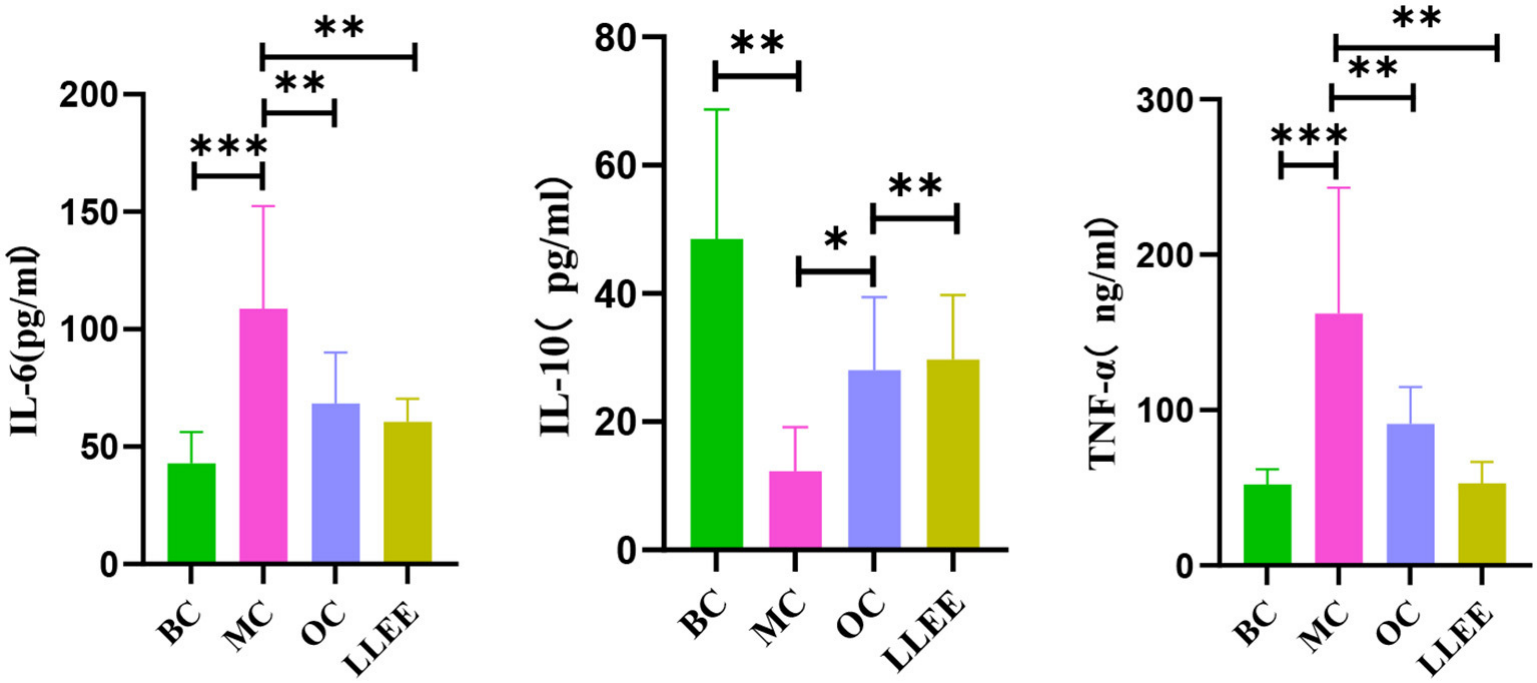
The levels of serum inflammatory factors of rats in each group. ^*^P < 0.05, ^**^P < 0.01, ^***^P < 0.001.

The serum expression of inflammatory factors of the rats in each group during the experiment is shown in Table 2. Enzyme-linked immunoassay kit was used to detect the expression of serum inflammatory factors. Compared with the BC group, the levels of a pro-inflammatory factor (IL-6, TNF-α) were significantly increased in the MC, OC, and LLEE groups, while the levels of an anti-inflammatory factor (IL-10) were significantly decreased, with a significant difference among groups (*p* < 0.01). Compared with the Orlistat capsule, LLEE significantly reduced the expression of the proinflammatory factor TNF-α (*p* < 0.01), while the expression of the anti-inflammatory factor IL-10 showed no significant difference (Figure 6). It was demonstrated that LLEE reduces the incidence of obesity by decreasing the expression of TNF-α.

**Table 2:** The levels of serum inflammatory factors of rats in each group.

**Group**	**TNF-α (*N* = 10) (ng/ml)**	**IL-6 (*N* = 10) (pg/ml)**	**IL-10 (*N* = 10) (pg/ml)**
BC	70.60 ± 22.70^**^	42.86 ± 13.32^**^	48.53 ± 20.15^**^
MC	142.72 ± 97.61^##^	108.70 ± 43.69^##^	12.32 ± 6.87^##^
OC	91.14 ± 23.72^**^	68.39 ± 21.70^#**^	28.02 ± 11.42^##**^
LLEE	60.07 ± 26.02^**^	64.48 ± 16.27^**^	29.68 ± 10.07^##**^

^**^P < 0.01, ^#^*P* < 0.05, ^##^*P* < 0.01.

### 3.8. Intestinal flora test results

To examine the species composition of each sample, OTU clustering was performed with a consistent 0.97 valid data for all samples, and the sequence of OTUs was specifically annotated. The data analysis discussed in the following subsections is based on the analysis of OTUs clustering results.

#### 3.8.1. Species abundance clustering heat map

**Figure 7:**
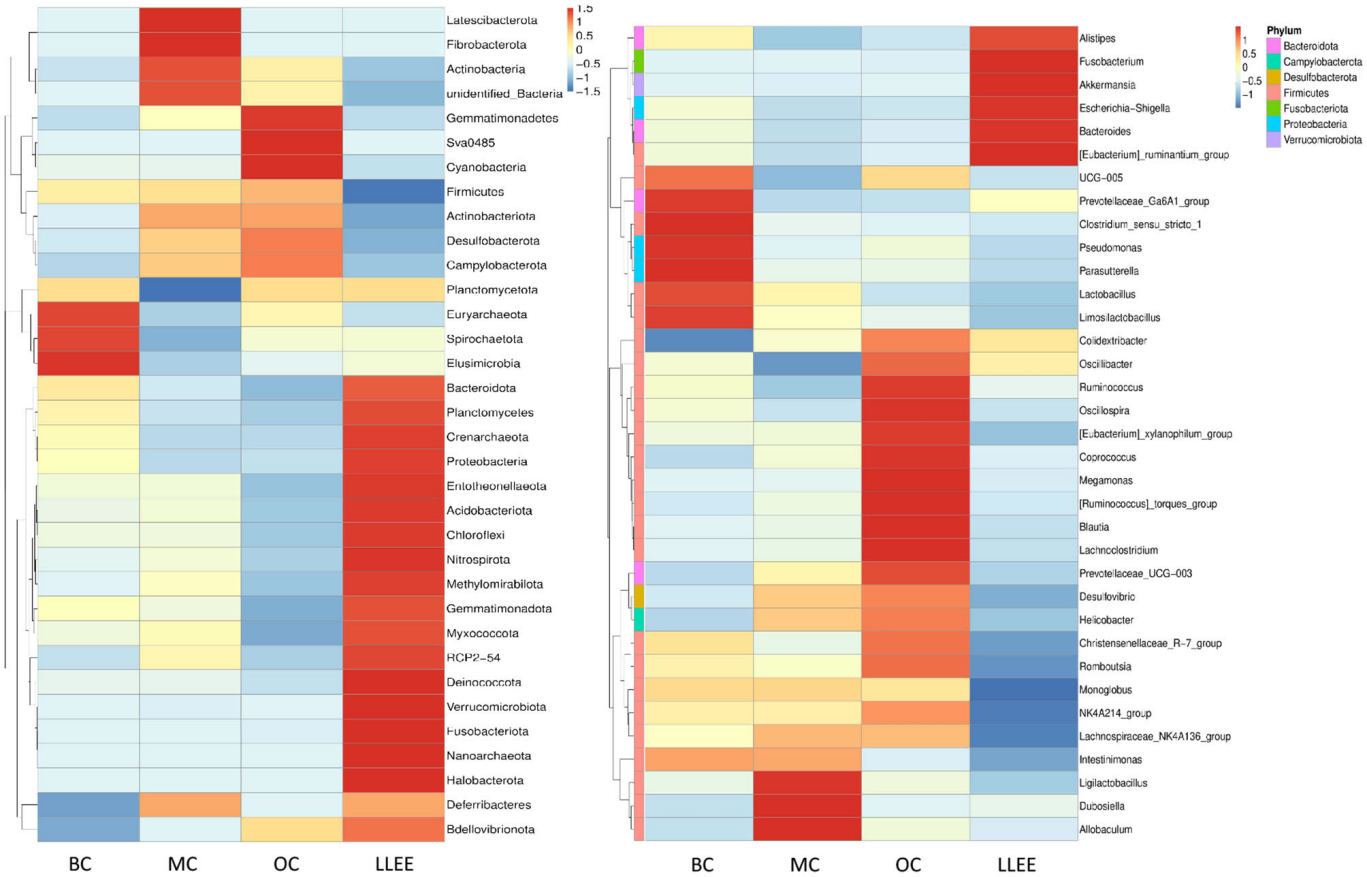
The level of species abundance clustering heat map at the phylum and genus levels.

According to the species annotation and abundant information of all samples at the phylum and genus levels, the top 35 genera with the highest abundance were selected. According to their abundance values in each sample, clustering was carried out from the two levels of species and samples. The heat map was drawn as shown in Figure 7, which depicts the discovery of species with high aggregation or low content in the samples. The horizontal bands shown in the figure are sample information. The species annotation information is on the right, and the clustering tree on the left is a species clustering tree. The depth of color indicates the abundance of the species.

The abundance of different bacterial species in each group and the variation trend of bacterial abundance were analyzed. The *t*-test was used to test the species groups, and significant differences were found in each classification level (*p* < 0.05). Compared with the BC group, the content of *Bacteroidota* in the MC group was significantly decreased (*p* < 0.05). The ratio of *Firmicutes* and *Bacteroidota* in the LLEE group recovered to the level of the BC group after administration (*p* < 0.05). The abundance of *Proteobacteria* and *Verrucomicrobiota* showed an increasing trend. The content of *Actinobacteria* and *Desulfobacterota* is decreased (*p* < 0.05). *Euryarchaeota* expression could be significantly decreased in the OC group (*p* < 0.05). At the genus level, it was shown that the imbalance of intestinal flora caused by a high-fat diet was concentrated in *Firmicutes*. The abundance of *Limosilactobacillus* and *Candidatus Saccharimonos* may have decreased due to a high-fat diet. *Colidextribacter, Lachnoclostridium, Blautia, Faecalibaculum*, and *Holdemania* showed an increasing trend in the MC group. For the OC group, a strong strain abundance fluctuation was caused. Although the same sample had a certain degree of microbial imbalance ability, its protective ability was obviously inferior to that of the LLEE group. In terms of species-level differences, both LLEE and Orlistat capsules achieved species-level protection for beneficial bacteria. However, the Orlistat capsule caused some species abundance fluctuations, which might be related to drug side effects.

#### 3.8.2. Comparative analysis of alpha diversity

The analysis of alpha diversity was used to analyze the microbial community diversity within the sample. The diversity analysis of a single sample can reflect the richness and diversity of the microbial community within the sample. The alpha diversity indices (Chao1, Ace, Shannon, Simpson, PD_whole_tree) of samples in each group under the consistency threshold of 0.97 was statistically analyzed. The results are shown in Table 3.

**Table 3:** Comparative analysis results of alpha diversity.

**Group**	**Observed_species**	**Chao1**	**Ace**	**Shannon**	**Simpson**	**PD_whole_tree**
BC	1,097.86 ± 128.35	1,199.62 ± 163.81	1,202.62 ± 144.07	7.70 ± 0.25	0.99 ± 0.00	54.14 ± 9.43
MC	1,057.86 ± 57.93	1,143.57 ± 75.35	1,157.42 ± 71.78	7.45 ± 0.38	0.98 ± 0.01	47.70 ± 2.97
OC	1,104.71 ± 110.97	1,182.97 ± 122.66^*^	1,206.93 ± 119.98	7.36 ± 0.28	0.98 ± 0.00^*^	51.66 ± 9.53^*^
LLEE	1,215.57 ± 64.51^#**⋆^	1,328.29 ± 90.28^**⋆^	1,343.29 ± 61.41^#**⋆^	6.75 ± 0.99^##*^	0.93 ± 0.07^#*⋆^	65.40 ± 3.01^##**⋆^

^*^P < 0.05, ^**^P < 0.01, ^#^*P* < 0.05, ^##^*P* < 0.01, ^⋆^*P* < 0.05, ^⋆⋆^*P* < 0.01.

#### 3.8.3. Comparative analysis of beta diversity

**Figure 8:**
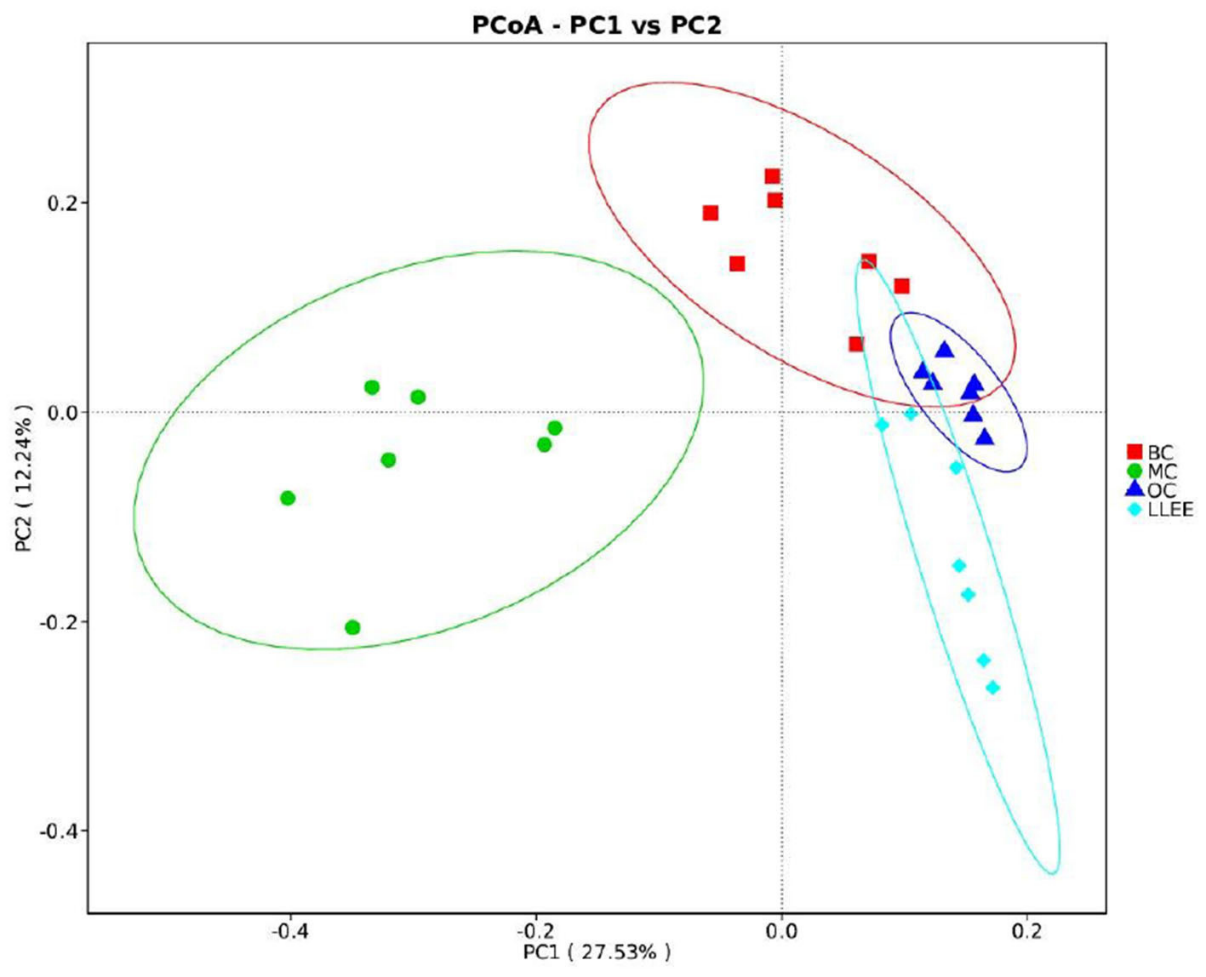
The characterization results of beta diversity.

Principal coordinates analysis (PCoA) analysis was used to extract the most important elements and structures from multidimensional data through a series of eigenvalues and eigenvector ordering. PCoA analysis was carried out based on Bray–Curtis distance and the principal coordinate combination with the largest contribution rate was selected for graph display. It is generally believed that the closer the distance between samples, the more similar the species composition structure. As a result, samples with highly similar community structures tend to cluster together, while samples with very different communities are separated far apart. The characterization results are shown in Figure 8.

#### 3.8.4. LEfSe analysis

**Figure 9:**
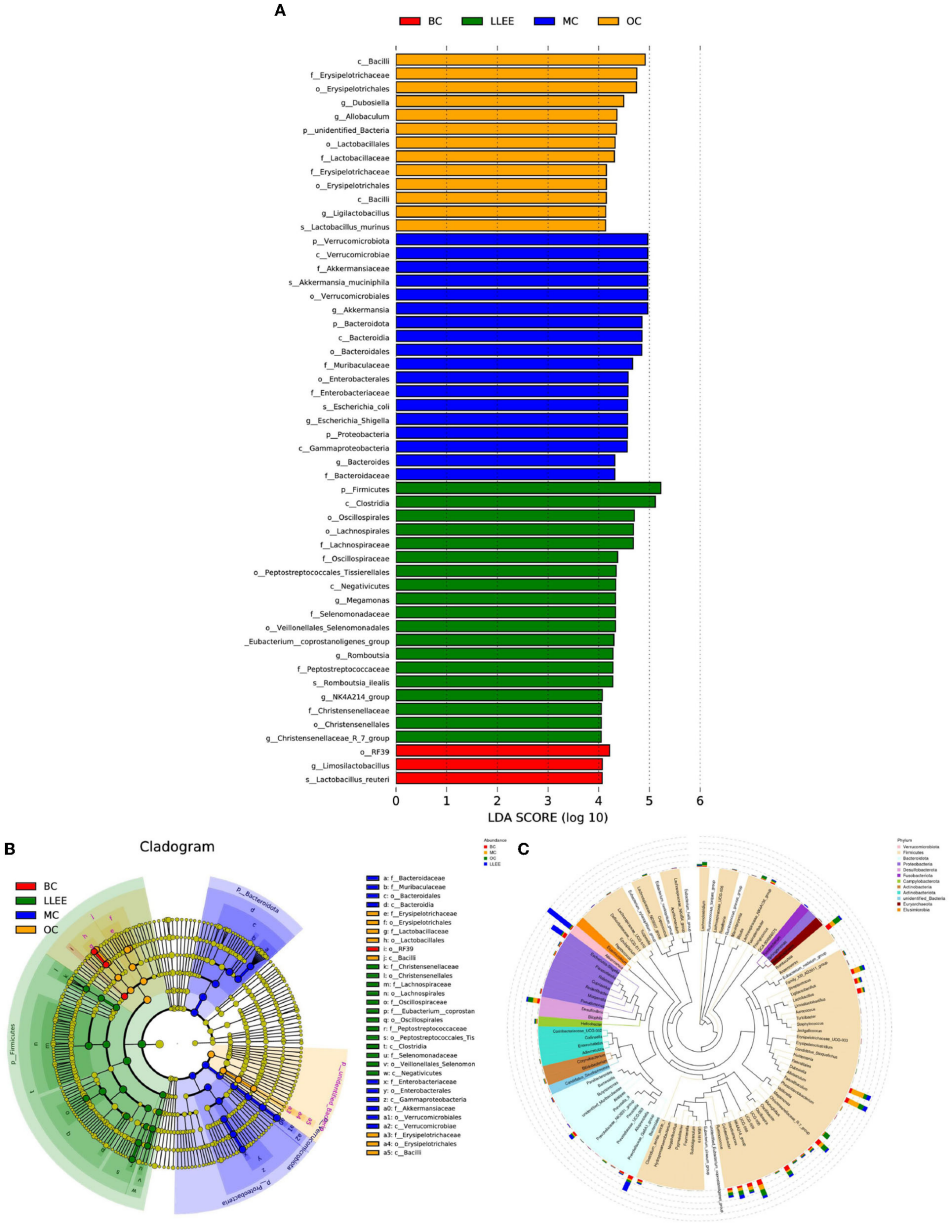
**(A–C)** The result of LEfSe analysis and the genus-level evolutionary tree.

LEfSe can directly analyze classification levels for statistical tests and difference analysis. Meanwhile, LEfSe placed more emphasis on finding stable biomarkers between groups. It is not limited to the analysis of community composition differences in different sample groups but can also go deep into different subgroups to select the signature microbial groups that show consistent performance in different subgroups. The analysis results showed that a high-fat diet had an effect on *Firmicutes, Christensenellaceae, Lachnospirales, Oscillospiraceae, Veillonellales-Selenemon, Eubacterium coprostan, Peptostreptococcaceae, Enterobacteriaceae, Akkermansiaceae, Bacteroidaceae, Muribaculaceae, Erysipelotrichaceae*. Moreover, LLEE mainly affects the abundance of *Bacilli, Erysipelotrichaceae, Lactobacillaceae, Limosilactobacillus, Lactobacillus reuteri, Clostridia*, and *Oscillospirales*. The amount of *Lachnospirales, Enterobacteriaceae, Gammaproteobacteria, Firmicutes, and Clostridia* was influenced with the Orlistat capsule (Figures 9A–C).

Although there were great individual differences in the composition of microbial flora, it was mainly composed of *Bacteroidetes* and *Firmicutes*. The dietary composition has an important effect on intestinal flora, and the relative proportion of these bacteria varies depending on diet. Diet cannot improve the body's condition without the mediation of intestinal flora (15–17). In this study, it was found that the abundance of *Bacteroidetes* increased, while the abundance of *Firmicutes* decreased in the cecum contents of rats on a high-fat diet. The LLEE intervention reversed the distribution of dominant flora abundance in the high-fat diet to some extent, indicating that the abundance ratio of *Bacteroidetes* and *Firmicutes* played an important role in the process of lipid-lowering (18, 19). LLEE protects against changes in the microbiota structure that are caused by fat intake. LLEE significantly reduced the abundance of *Brautella*, indicating that the liver weight and liver index of rats increased due to a high-fat diet. The damage to liver morphology and structure may be related to the increased abundance of *Brautella*. The LLEE intervention reduced the abundance of *Brautella* and improved liver damage. An experiment found that the abundance of *Brautzia* in patients with non-alcoholic steatohepatitis was significantly higher than that in healthy subjects, and the increase in abundance was in response to the intake of a high-fat diet (20). The results of this study also showed that LLEE significantly increased the abundance of *Parabacteroides* and decreased the abundance of *Prevoides*. It has been found that the abundance of *Parabacteroides* is higher in rats eating cellulose, and the abundance of *Parabacteroides* is negatively correlated with human body mass index (BMI) (21–23). The study showed that *Prevotella* mediates mucosal inflammation, inducing disease and inflammatory features by increasing the release of inflammatory mediators in immune cells and various stromal cells (24, 25). The results of this study showed that the abundance of *Parabacteroides* increased after LLEE intervention, while the abundance of *Prevoella* decreased. The study demonstrated that LLEE may play a preventive role in preventing obesity in rats fed a high-fat diet by upregulating the abundance of the beneficial bacterium *Parabacteroides* and downregulating the abundance of the proinflammatory bacterium *Prevosa*.

## 4. Discussion

The results of this study showed that LLEE could significantly reduce the body mass, Lee index, visceral WAT content, and serum biochemical indices TC, TG, and LDL-C levels of obese rats. At the same time, the serum levels of pro-inflammatory factor IL-6 and TNF-α were significantly decreased, and the serum levels of anti-inflammatory factor IL-10 were significantly increased. The HE staining results of liver tissue in each group showed that a high-fat diet could lead to fatty degeneration of liver cells and small focal inflammatory cell infiltration, and LLEE could significantly improve the morphological changes of liver cells and inflammatory factor infiltration caused by a high-fat diet. Compared with the Orlistat capsule, LLEE was more effective in regulating the composition and abundance of beneficial microbial flora in intestinal microbes, providing experimental data for regulating intestinal flora through dietary intervention.

LLEE can promote liver metabolism to some extent. High-throughput sequencing analysis of intestinal flora demonstrated that LLEE can increase the diversity of intestinal flora in rats with high-fat diets and cause changes in the intestinal flora structure. High-fat diet increased the abundance of *Bacteroidetes*, which was positively correlated with fat content, and of *Brautella*, which was associated with hepatitis, resulting in increased liver index and liver structural damage (26, 27). After LLEE intervention, liver disease caused by high-fat diet intake was significantly alleviated, and *Prevoella* was downregulated to alleviate inflammation caused by a high-fat diet. LLEE may play a preventive role against obesity in rats with high-fat diets by upregulating the abundance of the beneficial bacterium *Parabacteroides* and downregulating the abundance of the proinflammatory bacterium *Prevoella*.

The results of this study indicate that LLEE can not only regulate blood lipids and relieve chronic inflammation but also prevent antiobesity. It also provides a theoretical basis for dietary intervention to regulate intestinal flora and improve body health.

## Data availability statement

The original contributions presented in the study are included in the article/supplementary material, further inquiries can be directed to the corresponding author/s.

## Ethics statement

The animal study was reviewed and approved by Heilongjiang Academy of Chinese Medicine Science (No. 2015-64).

## Author contributions

ZY and ML: conceive ideas, complete experiments, data collection, arrangement, animal experiment implementation, and article revision. HJ: supportive work. WW: design and fund experiments. All authors contributed to the article and approved the submitted version.
